# In vivo 3D tomography of the lumbar spine using a twin robotic X-ray system: quantitative and qualitative evaluation of the lumbar neural foramina in supine and upright position

**DOI:** 10.1007/s00330-020-07355-x

**Published:** 2020-10-29

**Authors:** Anna L. Falkowski, Balazs K. Kovacs, Robyn M. Benz, Patrick Tobler, Stephan Schön, Bram Stieltjes, Anna Hirschmann

**Affiliations:** 1 Department of Radiology, Clinic of Radiology and Nuclear Medicine, University Hospital Basel, University of Basel, Petersgraben 4, 4031 Basel, Switzerland; 2 Department of Radiology, Orthopedic University Hospital Balgrist, University of Zurich, Forchstrasse 340, 8008 Zurich, Switzerland; 3 Department of Radiology, McGill University Health Center, 1001 Decarie Blvd, Montreal, Quebec, H4A 3 J1 Canada; 4 Clinic for Spinal Surgery, University Hospital Basel, University of Basel, Petersgraben 4, 4031 Basel, Switzerland

**Keywords:** Radiologic technology, Tomography, Weight-bearing, Spine, Back pain

## Abstract

**Objectives:**

Supine lumbar spine examinations underestimate body weight effects on neuroforaminal size. Therefore, our purpose was to evaluate size changes of the lumbar neuroforamina using supine and upright 3D tomography and to initially assess image quality compared with computed tomography (CT).

**Methods:**

The lumbar spines were prospectively scanned in 48 patients in upright (3D tomographic twin robotic X-ray) and supine (30 with 3D tomography, 18 with CT) position. Cross-sectional area (CSA), cranio-caudal (CC), and ventro-dorsal (VD) diameters of foramina were measured by two readers and additionally graded in relation to the intervertebral disc height. Visibility of bone/soft tissue structures and image quality were assessed independently on a 5-point Likert scale for the 18 patients scanned with both modalities. Descriptive statistics, Wilcoxon’s signed-rank test (*p* < 0.05), and interreader reliability were calculated.

**Results:**

Neuroforaminal size significantly decreased at all levels for both readers from the supine (normal intervertebral disc height; CSA 1.25 ± 0.32 cm^2^; CC 1.84 ± 0.24 cm^2^; VD 0.88 ± 0.16 cm^2^) to upright position (CSA 1.12 ± 0.34 cm^2^; CC 1.78 ± 0.24 cm^2^; VD 0.83 ± 0.16 cm^2^; each *p* < 0.001). Decrease in intervertebral disc height correlated with decrease in foraminal size (supine: CSA 0.88 ± 0.34 cm^2^; CC 1.39 ± 0.33 cm^2^; VD 0.87 ± 0.26 cm^2^; upright: CSA 0.83 ± 0.37 cm^2^, *p* = 0.010; CC 1.32 ± 0.33 cm^2^, *p* = 0.015; VD 0.80 ± 0.21 cm^2^, *p* = 0.021). Interreader reliability for area was fair to excellent (0.51–0.89) with a wide range for cranio-caudal (0.32–0.74) and ventro-dorsal (0.03–0.70) distances. Image quality was superior for CT compared with that for 3D tomography (*p* < 0.001; *κ*, CT = 0.66–0.92/3D tomography = 0.51–1.00).

**Conclusions:**

The size of the lumbar foramina is smaller in the upright weight-bearing position compared with that in the supine position. Image quality, especially nerve root delineation, is inferior using 3D tomography compared to CT.

**Key Points:**

*• Weight-bearing examination demonstrates a decrease of the neuroforaminal size.*

*• Patients with higher decrease in intervertebral disc showed a narrower foraminal size.*

*• Image quality is superior with CT compared to 3D tomographic twin robotic X-ray at the lumbar spine.*

## Introduction

Direct and indirect costs of low back pain cause a tremendous financial burden to society [1]. Therefore, cross-sectional examinations of the lumbar spine are common in addition to radiographs. These cross-sectional examinations are generally performed in the supine non weight-bearing position. This is because upright magnetic resonance imaging (MRI) scanners, which allow weight-bearing examinations, are not widely available [2] and weight-bearing computed tomography (CT) examinations are only feasible for the lower extremities, but not for the spine [3, 4].

It is known that leg pain due to a lumbar foramen stenosis is more pronounced in the upright position [5]. Therefore, radiologic images, obtained in a supine position, do not necessarily represent the true extent of pathology [2]. This discrepancy is a challenge for diagnosis and therapy, especially if clinical symptoms and imaging findings differ. Studies have shown that the size of the lumbar foramina decreases and stenosis increases with weight-bearing [6–14]. However, these studies were performed either in a seated position [8, 13, 15–18] or in simulated weight-bearing in supine position by applying axial loading of 40–50% of the body weight with a compressive device—the so-called axial loaded supine MR technique [2, 19–21]. Also, nearly vertical examinations in an approximately 80° upright position were performed [22, 23]. However, none of these studies assessed the differences of the lumbar foramina in a physiological upright weight-bearing and a supine position. A lately introduced multifunctional X-ray system with a twin robotic X-ray technology enables 3D tomography of the lumbar spine in the supine and upright position. However, only a phantom study, a cadaveric study of the lumbar spine, and in vivo studies of the extremities evaluated the system so far [24–28].

The purpose of this study was to quantitatively evaluate lumbar neuroforaminal size differences between the supine non-weight-bearing and the upright weight-bearing positions. Furthermore, an initial assessment of image quality using 3D tomography in comparison with CT of the lumbar spine was perused.

## Materials and methods

### Patients and demographics

All patients were prospectively enrolled after Institutional Review Board approval. Informed consent was given. Patients referred for lumbar spine imaging (radiographs or CT) from the spine center, orthopedics, or the emergency department were included consecutively by the musculoskeletal radiology department in a period of 8 months from February to September 2016. All patients were examined in the upright position with 3D tomography. Patients referred for upright radiographs also received an upright and supine 3D tomography, whereas those referred to supine CT additionally received an upright 3D tomography. Thus, an upright and a supine scan of each patient was performed. Patients with age < 18 years or inability to maintain a standing position were excluded. Stabilized lumbar segments were excluded from analysis due to metallic artifacts at these segments and lack of precise area determination after foraminotomy and laminectomy. Abnormal anatomic segmentation was not an exclusion criterion.

Forty-eight patients (14 males; 34 females) with a mean age of 68 ± 13 years were included in the study. No abnormal anatomic segmentation was observed in these patients. 3D tomography of the lumbar spine in the upright weight-bearing position was performed in all included patients. Thirty patients were examined in both the supine and upright positions using 3D tomography. Eighteen patients were examined in the supine position using a multislice CT scanner. Initial reasons for imaging of the included patients were as follows: follow-up after lumbar spine fusion surgery (*n* = 27), low back pain (*n* = 9), trauma (*n* = 5), spondylolisthesis (*n* = 4), or spinal canal stenosis (*n* = 3). The lumbar fusion surgery of the included patients was performed 1 week to 11 years prior to our study exams. In total, 480 neural foramina were included; of these, 118 were excluded according to the established criteria, and 362 were finally analyzed. In detail, bilaterally 84 neural foramina at the level L1 were included, 86 at L2, 78 at L3, 58 at L4, and 56 at L5.

### Imaging technique

A twin robotic X-ray unit (Multitom Rax, Siemens Healthineers) was used to acquire 3D tomographies of the extremities. For upright scans, the following parameters were used: projections, 160; tube voltage, 81–121 kVp; tube current, 178.6–1671.9 mAs; with and without a copper filter of 0.2 mm thickness; scan time, 20 s; scan range, 23 cm. The supine non-weight-bearing scan using 3D tomography had the following parameters: projections, 160; tube voltage, 109–125 kVp; tube current, 208.1–1116.98 mAs; with and without a copper filter of 0.2 mm thickness; scan time, 20 s; scan range, 23 cm. Image reconstruction algorithm was a very smooth kernel. Dose–length product for 3D tomography in the supine position was in mean 644 mGy*cm, and 410 mGy*cm in the upright position.

CT examinations (Somatom AS+, Somatom Definition Edge, Somatom Definition Flash; Siemens Healthineers) were performed with clinical-appropriated scan protocols: tube voltage, 100–120 kVp; tube current, 125–266 mAs; pitch factor, 0.8; matrix, 512 × 512; reconstruction thickness, 0.75 mm; reconstruction increment, 0.5 mm; scan time, 8–30 s; scan range, 24–50 cm; automatic tube current modulation (CareDose4D, Siemens Healthineers). An iterative reconstruction (SAFIRE 3) was used for image reconstruction for the CT. Dose–length product for supine CT examinations was in mean 438 mGy*cm. In comparison, the dose–length product of the same patients using 3D tomography in the upright position was in mean 400 mGy*cm.

### Quantitative image analysis

**Fig. 1:**
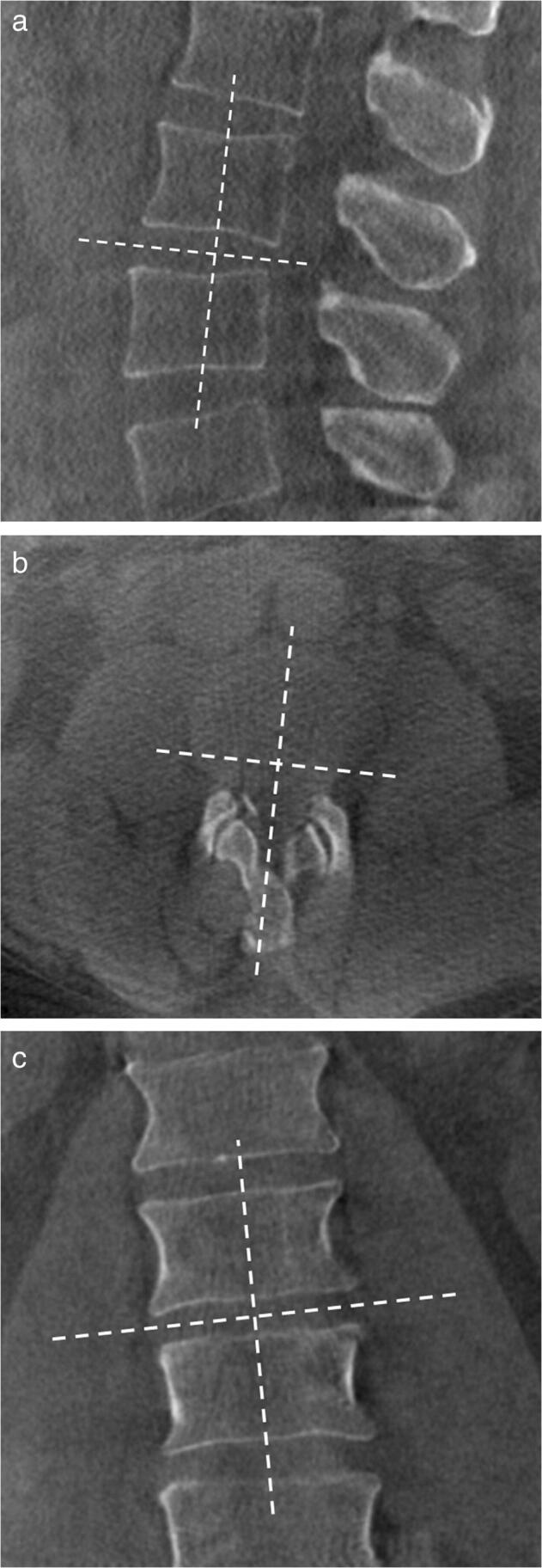
Adjustment of the axes on all imaging planes along the intervertebral disc space in the lumbar spine of a 42-year-old male in the (**a**) sagittal, (**b**) axial, and (**c**) coronal planes indicated by dashed lines

Images were extracted from the PACS system and analyzed anonymously using OsiriX (Pixmeo). Interpretation tools, e.g., magnification and contrast, were available to use. 3D tomographic and CT datasets were evaluated on 3D multiplanar bone window reconstructions with a slice thickness of 3 mm. Images were reformatted in alignment to the intervertebral disc and axis of the spine at each lumbar level (Fig. 1). Measurements were performed by two independent readers with 1 and 5 years of experience in musculoskeletal radiology. Intervertebral disc height on tomographies performed in the upright position was graded on each site of the neural foramina (0 = normal; 1 = narrowing with > 50% height of the intervertebral space preserved; 2 = narrowing with < 50% height of the intervertebral space preserved). Size of the neural foramina (area; cranio-caudal and ventro-dorsal diameters) was measured on sagittal reformats at the section with the narrowest cross-sectional area. The cross-sectional area was defined by the osseous borders of the neural foramina. The cranio-caudal diameter was measured at the largest distance between the inferior margin of the pedicle of the superior vertebra and the superior margin of the pedicle of the inferior vertebra. The ventro-dorsal diameter was measured perpendicular to the cranio-caudal measurement at the largest distance.

### Qualitative image analysis

3D tomographic and CT datasets were evaluated on 3D multiplanar soft tissue reconstructions with a slice thickness of 3 mm. For image analysis, the OsiriX image processing software was used. Visibility of right and left neural nerve roots, flava ligaments, and image quality (artifacts, noise, overall image quality) were assessed on a 5-point Likert scale independently by two readers with 1 and 8 years of experience in musculoskeletal radiology for the 18 patients scanned with both modalities in the supine position. The Likert scale was encoded for artifacts as follows: 1 = no artifacts, 2 = minor artifacts without influence on image assessment, 3 = moderate artifacts without influence on image assessment (diagnosis still possible), 4 = major artifacts with influence on image assessment (diagnosis impaired), and 5 = severe artifacts making diagnosis impossible; and for all other items as follows: 1 = excellent, 2 = good, 3 = fair, 4 = poor, 5 = inadequate. Qualitative image analysis was performed at levels L1, L3, and L5.

### Statistical analysis

Descriptive statistics were used to report the quantitative and qualitative data. The Wilcoxon signed-rank test was utilized to assess significant differences between scans in the supine and upright positions and to analyze image quality. A *p* value less than 0.05 was considered statistically significant. Interreader reliability was defined by intraclass correlation coefficient (ICC) for quantitative measurements and Cohen’s kappa for qualitative evaluation. According to Rosner for the interreader reliability, an ICC value of > 0.75 is considered excellent, 0.40–0.75 fair to good, and < 0.40 poor [29]. For the interreader reliability with Cohen’s kappa (*κ*), a value of 0.81–1.00 is considered (almost) perfect, 0.61–0.80 substantial, 0.41–0.60 moderate, 0.21–0.40 fair, 0–0.20 slight, and < 0 poor [30]. All statistics were performed using the SPSS software (version 22.0).

## Results

### Quantitative analysis

**Fig. 2:**
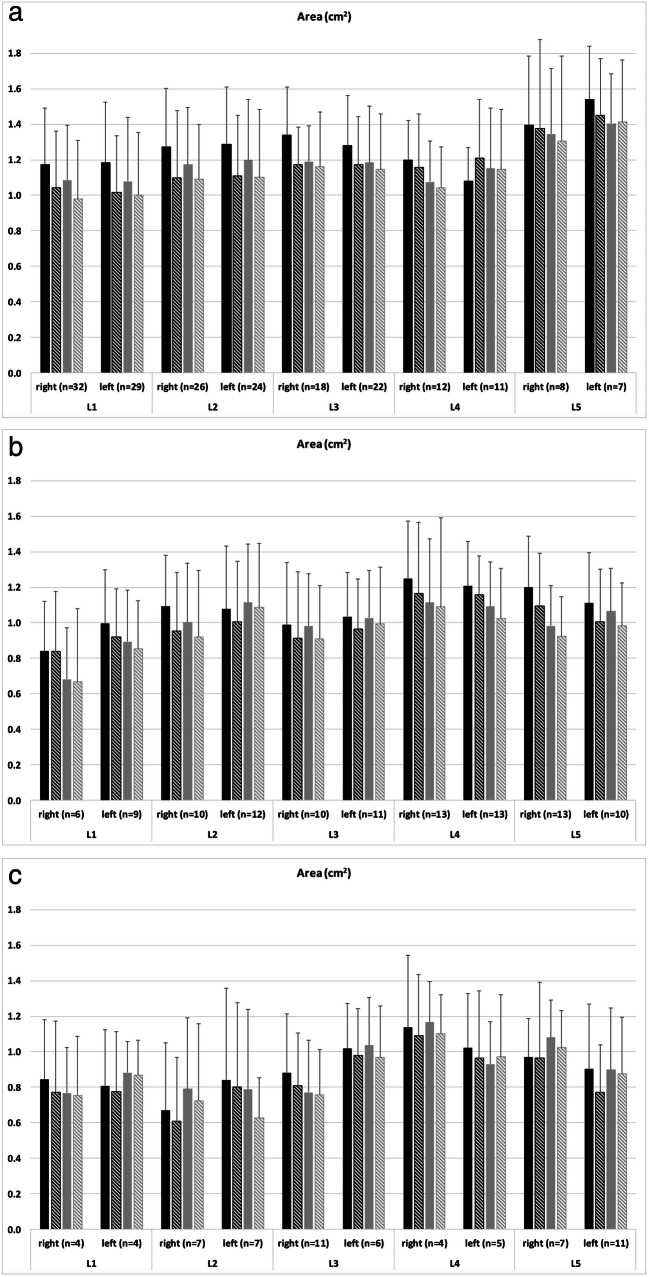
Histograms present the measurements of the cross-sectional area of all included neural foramina of both readers and both sides (**a**) with normal intervertebral height, (**b**) narrowing with > 50% height of the intervertebral space preserved, and (**c**) narrowing with < 50% height of the intervertebral space preserved. The black columns represent reader 1 and the gray columns represent reader 2. The filled columns show the supine position and the hatched columns the upright position. Data are mean values with standard deviations

**Fig. 3:**
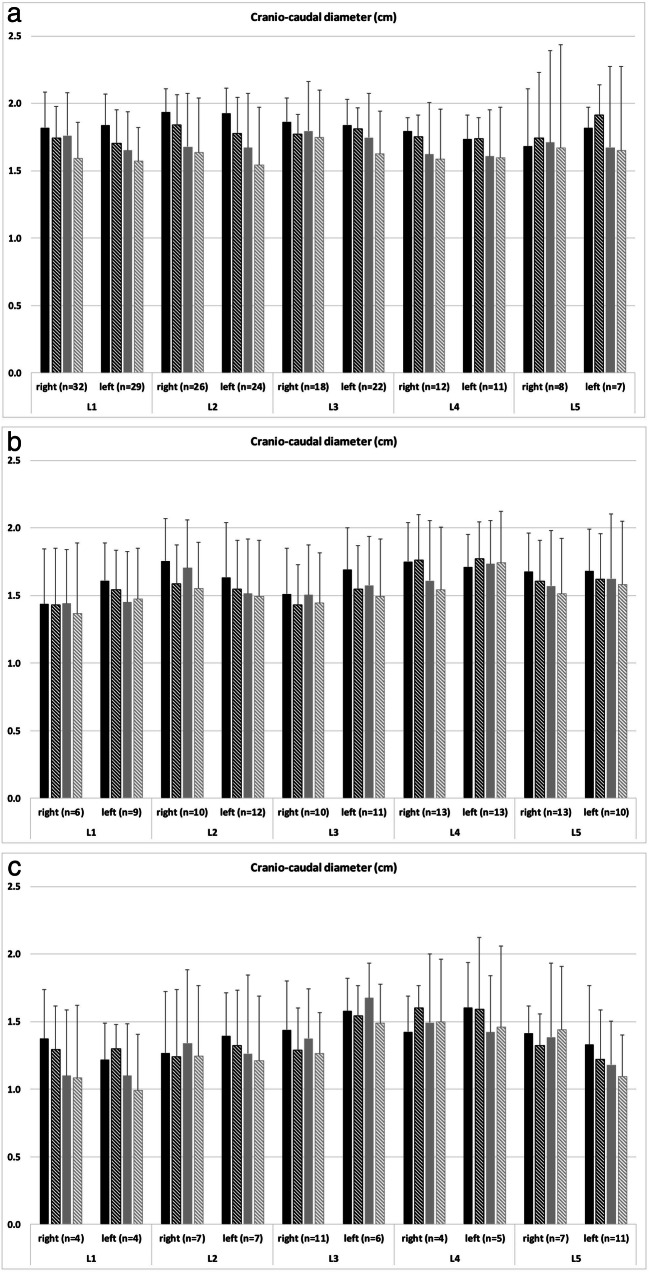
Histograms present the measurements of the cranio-caudal diameter of all included neural foramina of both readers and both sides **a** with normal intervertebral height, **b** narrowing with > 50% height of the intervertebral space preserved, and **c** narrowing with < 50% height of the intervertebral space preserved. The black columns represent reader 1 and the gray columns represent reader 2. The filled columns show the supine position and the hatched columns the upright position. Data are mean values with standard deviations

**Fig. 4:**
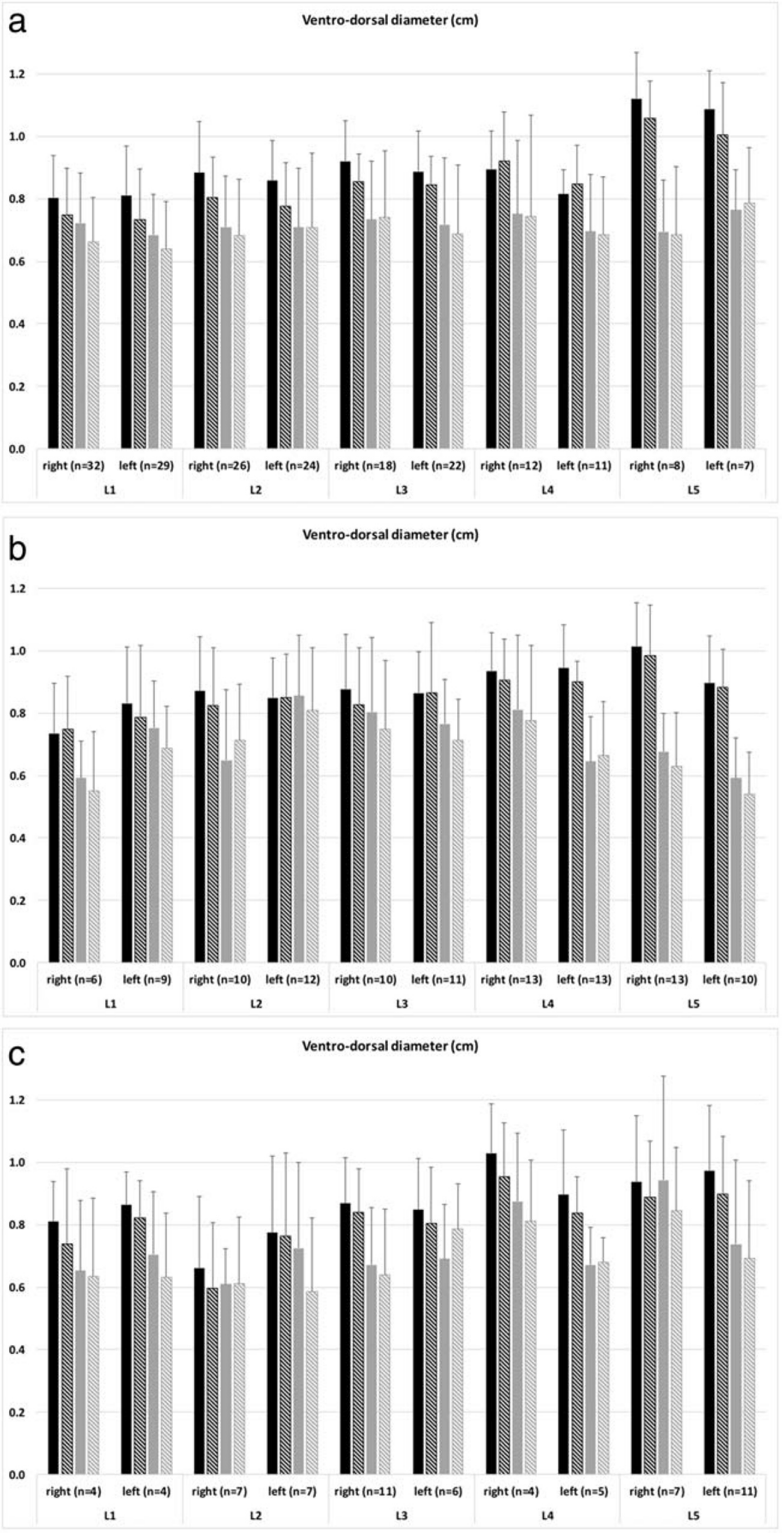
Histograms present the measurements of the ventro-dorsal diameter of all included neural foramina of both readers and both sides **a** with normal intervertebral height, **b** narrowing with > 50% height of the intervertebral space preserved, and **c** narrowing with < 50% height of the intervertebral space preserved. The black columns represent reader 1 and the gray columns represent reader 2. The filled columns show the supine position and the hatched columns the upright position. Data are mean values with standard deviations

**Fig. 5:**
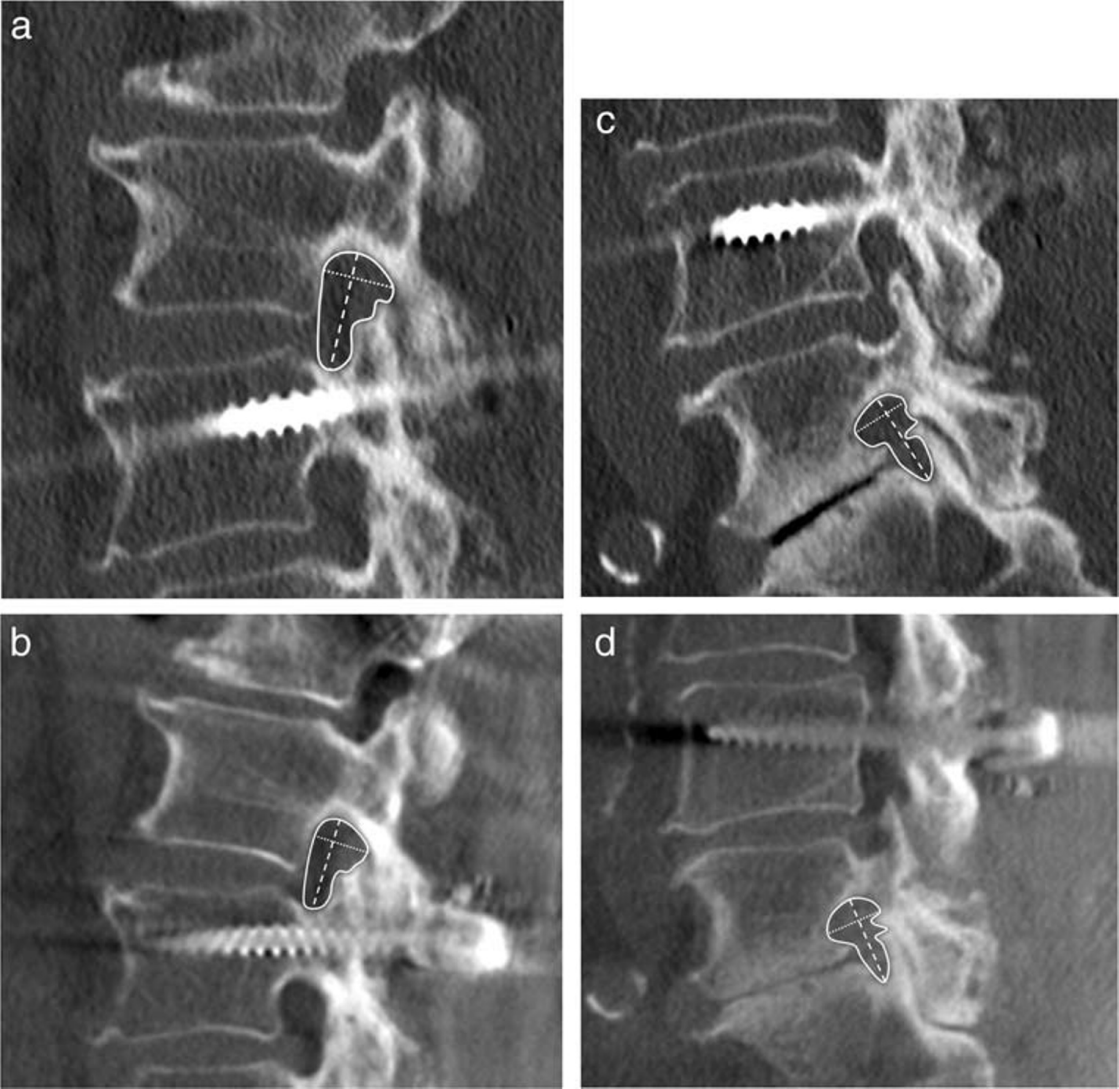
Comparison of the right L2 and L5 neural foramen in a 68-year-old female on supine CT (**a**/**c**) and upright 3D tomography (**b**/**d**). L2 neural foramen with normal intervertebral disc height shows a decrease in size from 1.29 cm^2^ for cross-sectional area (continuous line), 1.8 cm for cranio-caudal (dashed line), and 1.1 cm for ventro-dorsal (dotted line) diameters in the non-weight-bearing position (**a**) to 1.20 cm^2^, 1.7 cm cranio-caudal, and 1.0 cm ventro-dorsal in the weight-bearing position (**b**), respectively. The size of the L5 neural foramen is unchanged for cranio-caudal (1.69 cm) and ventro-dorsal diameters (1.0 cm) but shows a slight reduction of the cross-sectional area from 1.04 cm^2^ in the non-weight-bearing to 0.94 cm^2^ in the weight-bearing position (**c**, **d**) due to preexisting severe osteochondrosis

In total, the cross-sectional area of the neural foramina decreased significantly at nearly all levels for both readers from the supine (e.g., reader 1; all right lumbar segments with normal intervertebral height: 1.25 ± 0.32 cm^2^) to upright (1.12 ± 0.34 cm^2^) position for all grades of intervertebral height changes (Fig. 2 and Table 1). In detailed observation of all levels separately, there were only exceptions for L4 left reader 1 (normal intervertebral height; Fig. 2a) and L4 left reader 2 (< 50% intervertebral height preserved; Fig. 2c). The cranio-caudal (e.g., reader 1; all right lumbar segments with normal intervertebral height: supine 1.84 ± 0.24 cm^2^; upright 1.78 ± 0.24 cm^2^) and ventro-dorsal (e.g., reader 1; all right lumbar segments with normal intervertebral height: supine 0.88 ± 0.16 cm^2^; upright 0.83 ± 0.16 cm^2^) diameters of the neural foramina also decreased significantly at nearly all levels for both readers from the supine to upright position (Figs. 3 and 4 and Table 1). Exceptions for detailed observation are shown in Fig. 3 for cranio-caudal and Fig. 4 for ventro-dorsal diameter changes. An example of the influence of intervertebral disc height changes on the neuroforaminal size is depicted in Fig. 5.

**Table 1:** Changes in neuroforaminal size (mean and standard deviation) for all lumbar segments combined (right or left side) between supine and upright positions in patients with different grading of intervertebral heights for all 48 patients

		Area (cm^2^)				Cranio-caudal diameter (cm)				Ventro-dorsal diameter (cm)			
		Reader 1		Reader 2		Reader 1		Reader 2		Reader 1		Reader 2	
		Supine	Upright	Supine	Upright	Supine	Upright	Supine	Upright	Supine	Upright	Supine	Upright
0 *n* = 189	Right	1.25 ± 0.32	1.12 ± 0.34	1.15 ± 0.30	1.08 ± 0.32	1.84 ± 0.24	1.78 ± 0.24	1.72 ± 0.39	1.64 ± 0.39	0.88 ± 0.16	0.83 ± 0.16	0.72 ± 0.17	0.69 ± 0.20
	*p* value	< 0.001*		< 0.001*		< 0.001*		< 0.001*		< 0.001*		< 0.001*	
	Left	1.25 ± 0.32	1.13 ± 0.33	1.17 ± 0.34	1.11 ± 0.35	1.85 ± 0.21	1.77 ± 0.23	1.67 ± 0.36	1.59 ± 0.36	0.86 ± 0.15	0.80 ± 0.15	0.70 ± 0.17	0.69 ± 0.20
	*p* value	< 0.001*		< 0.001*		< 0.001*		< 0.001*		< 0.001*		< 0.001*	
1 *n* = 107	Right	1.11 ± 0.33	1.02 ± 0.36	0.98 ± 0.32	0.93 ± 0.38	1.65 ± 0.33	1.58 ± 0.34	1.58 ± 0.40	1.50 ± 0.41	0.91 ± 0.17	0.88 ± 0.18	0.72 ± 0.21	0.70 ± 0.21
	*p* value	< 0.001*		< 0.001*		< 0.001*		< 0.001*		0.008*		0.001*	
	Left	1.10 ± 0.29	1.02 ± 0.29	1.05 ± 0.28	1.00 ± 0.30	1.67 ± 0.31	1.62 ± 0.32	1.60 ± 0.39	1.57 ± 0.41	0.88 ± 0.15	0.86 ± 0.16	0.72 ± 0.18	0.69 ± 0.18
	*p* value	< 0.001*		< 0.001*		0.003*		< 0.001*		0.057		0.003*	
2 *n* = 66	Right	0.88 ± 0.34	0.83 ± 0.37	0.88 ± 0.32	0.85 ± 0.32	1.39 ± 0.33	1.32 ± 0.33	1.35 ± 0.46	1.30 ± 0.43	0.87 ± 0.26	0.80 ± 0.21	0.74 ± 0.25	0.70 ± 0.22
	*p* value	0.010*		0.012*		0.015*		0.020*		0.021*		0.052	
	Left	0.92 ± 0.36	0.85 ± 0.34	0.90 ± 0.32	0.85 ± 0.30	1.42 ± 0.36	1.37 ± 0.38	1.32 ± 0.43	1.24 ± 0.43	0.88 ± 0.20	0.83 ± 0.19	0.71 ± 0.22	0.68 ± 0.20
	*p* value	0.004*		0.102		0.042*		0.015*		0.014*		0.195	

*p* values < 0.05 are marked with an asterisk

The grading of the intervertebral height was encoded as follows: 0 = normal; 1 = narrowing with > 50% height of the intervertebral space preserved; 2 = narrowing with < 50% height of the intervertebral space preserved

The ICC for the area was fair to excellent (0.50–0.89) at all levels, and at nearly all levels for the cranio-caudal diameter (0.32–0.85; Table 2). The ICC for the cranio-caudal diameter was poor for the supine position at the left L4 level (0.32) and the right L5 level (0.39). The ICC for the ventro-dorsal diameter showed a wide range (0.03–0.70; Table 2).

**Table 2:** Intraclass correlation coefficient (ICC) for interreader reliability of cross-sectional area, cranio-caudal, and ventro-dorsal diameters of neural foramen of all 48 patients

	Area				Cranio-caudal diameter				Ventro-dorsal diameter			
	Right		Left		Right		Left		Right		Left	
	Supine	Upright	Supine	Upright	Supine	Upright	Supine	Upright	Supine	Upright	Supine	Upright
L1 *n* = 84	0.87	0.89	0.88	0.85	0.60	0.65	0.74	0.68	0.56	0.70	0.57	0.55
L2 *n* = 86	0.70	0.72	0.80	0.76	0.67	0.69	0.71	0.70	0.11	0.57	0.39	0.40
L3 *n* = 78	0.84	0.88	0.81	0.88	0.67	0.74	0.48	0.54	0.46	0.43	0.35	0.35
L4 *n* = 58	0.57	0.75	0.72	0.50	0.55	0.63	0.32	0.58	0.30	0.38	0.35	0.13
L5 *n* = 56	0.71	0.74	0.85	0.84	0.39	0.48	0.50	0.57	0.03	0.07	0.31	0.26

### Qualitative image analysis

**Fig. 6:**
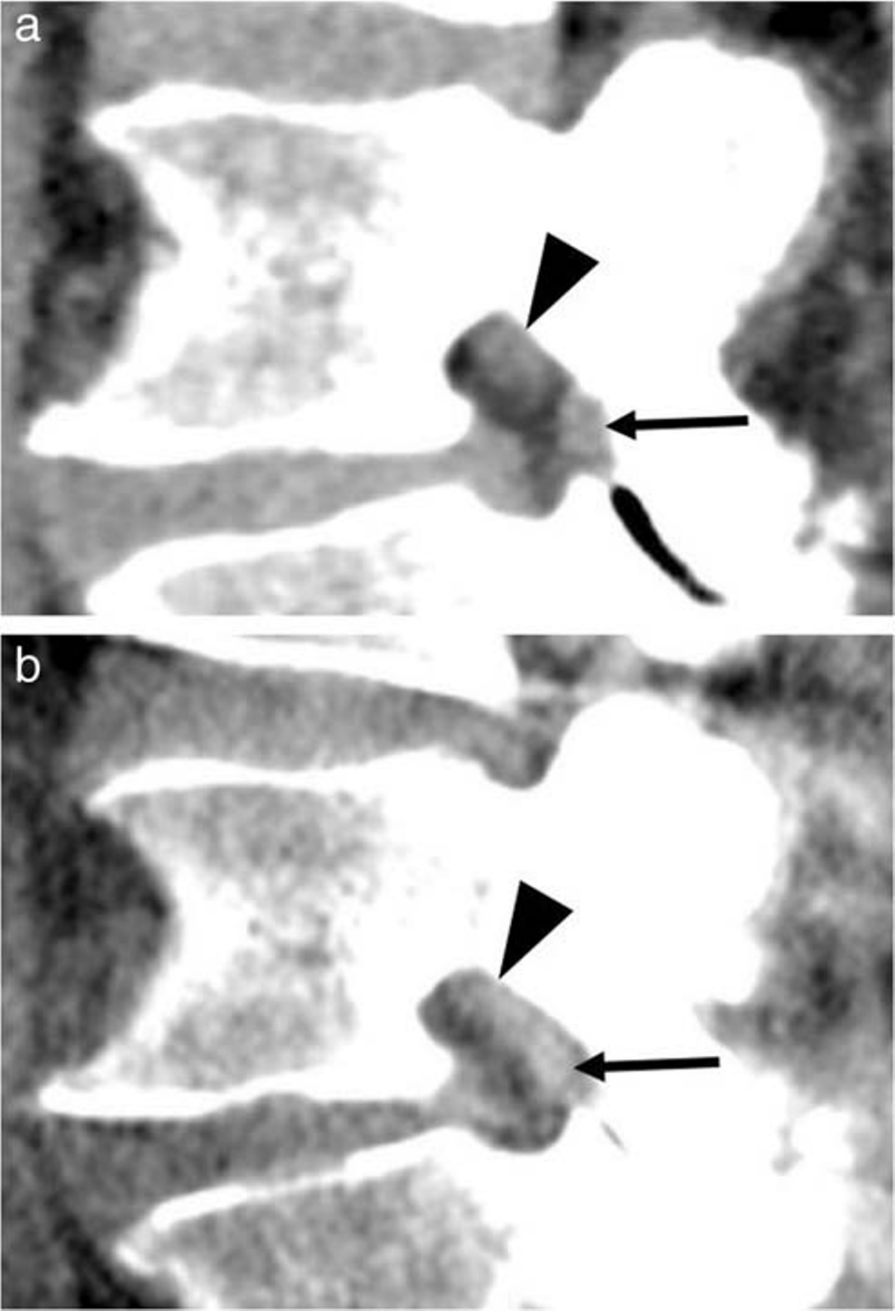
Comparison of image quality of soft tissue resolution of the neural foramen and flava ligaments on CT (**a**) and 3D tomography (**b**). Soft tissue resolution is better with CT. Note that contact of the flavum ligament (**b**; arrow) to the nerve root was depicted with weight-bearing. Therefore, perineural fat is obliterated to half of the nerve root circumference in standing (**b**; arrowhead) compared with three quarters of the circumference in the supine position (**a**; arrowhead). However, the nerve boundary is blurred on 3D tomography (**b**) compared with that on CT (**a**) representing the higher noise and reduced overall image quality using twin robotic X-ray

Image quality of the soft tissue was superior with CT compared to 3D tomography using twin robotic X-ray (*p* < 0.001) for both readers and all parameters (Fig. 6 and Table 3). In detail, both readers rated the visibility of left and right nerve roots as well as flava ligaments “fair” to “poor” on 3D tomography (mean, 3.2–4.0) and “excellent” to “good” on CT (mean, 1.0–1.9). Artifacts were evaluated as “moderate (without influence on image assessment)” on 3D tomography (mean, 3.0) and “no artifacts” were seen at CT (mean, 1.0–1.1), while noise and overall image quality were rated “fair” at 3D tomography (mean, 2.7–3.1) and “excellent” to “good” at CT (mean, 1.0–1.6). Interreader reliability was substantial to perfect for almost all qualitative parameters: detection of right and left nerve root (*κ* = 0.74–0.92 for 3D tomography; *κ* = 0.66–0.84 for CT), flava ligaments (*κ* = 0.76–0.89 for 3D tomography; *κ* = 0.73–0.84 for CT), assessment of artifacts (*κ* = 1.00 for 3D-tomography; *κ* = 0.92 for CT), and image quality (*κ* = 0.86 for 3D tomography; *κ* = 0.66–0.85 for CT). The agreement was moderate (*κ* = 0.51 for 3D tomography) and substantial (*κ* = 0.68 for CT) for the assessment of signal-to-noise ratio.

**Table 3:** Qualitative evaluation of soft tissue structures and overall image quality in the 18 patients examined with 3D tomography and CT

		**Visibility**											
		**Right neural nerve root**				**Left neural nerve root**				**Flava ligaments**			
		**Reader 1**		**Reader 2**		**Reader 1**		**Reader 2**		**Reader 1**		**Reader 2**	
		**3D**	**CT**	**3D**	**CT**	**3D**	**CT**	**3D**	**CT**	**3D**	**CT**	**3D**	**CT**
**L1**	**Mean ± sd**	3.6 ± 0.7	1.8 ± 0.9	3.7 ± 0.8	1.0 ± 0	3.3 ± 0.5	1.6 ± 1.1	3.5 ± 0.7	1.0 ± 0	3.6 ± 1.0	1.3 ± 0.5	3.7 ± 0.7	1.0 ± 0
	***p***	0.001*		0.001*		0.002*		0.001*		0.001*		0.001*	
**L3**	**Mean ± sd**	3.6 ± 0.8	1.9 ± 1.6	3.8 ± 0.9	1.2 ± 0.8	3.2 ± 0.8	1.9 ± 1.5	3.4 ± 0.8	1.3 ± 0.9	3.4 ± 1.0	1.9 ± 1.4	3.8 ± 0.9	1.1 ± 0.5
	***p***	0.001*		< 0.001*		0.005*		< 0.001*		0.001*		< 0.001*	
**L5**	**Mean ± sd**	3.8 ± 0.8	1.8 ± 1.5	4.0 ± 0.8	1.1 ± 0.5	3.9 ± 0.8	1.8 ± 1.1	4.0 ± 0.7	1.0 ± 0	3.9 ± 1.0	1.5 ± 1.0	3.7 ± 1.0	1.1 ± 0.3
	***p***	0.001*		0.001*		0.001*		< 0.001*		< 0.001*		< 0.001*	
		**Overall**											
		**Artifacts**				**Signal-noise ratio**				**Image quality**			
		**Reader 1**		**Reader 2**		**Reader 1**		**Reader 2**		**Reader 1**		**Reader 2**	
		**3D**	**CT**	**3D**	**CT**	**3D**	**CT**	**3D**	**CT**	**3D**	**CT**	**3D**	**CT**
**L1–L5**	**Mean ± sd**	3.0 ± 0	1.1 ± 0.3	3.0 ± 0	1.0 ± 0	3.0 ± 0.6	1.6 ± 0.6	2.7 ± 0.5	1.0 ± 0	3.1 ± 0.5	1.2 ± 0.4	3.0 ± 0.7	1.0 ± 0
	***p***	< 0.001*		< 0.001*		< 0.001*		< 0.001*		< 0.001*		< 0.001*	

*p* values < 0.05 are marked with an asterisk. *3D*, 3D tomography; *CT*, computed tomography; *sd*, standard deviation. The Likert scale was encoded for artifacts as follows: 1 = no artifacts, 2 = minor artifacts without influence on image assessment, 3 = moderate artifacts without influence on image assessment (diagnosis still possible), 4 = major artifacts with influence on image assessment (diagnosis impaired), and 5 = severe artifacts making diagnosis impossible; and for all other items as follows: 1 = excellent, 2 = good, 3 = fair, 4 = poor, 5 = inadequate

## Discussion

In our study, we found a significant decrease of neural foraminal height between supine and upright positions. Patients with narrower intervertebral disc height showed a narrower neural foraminal size in supine and upright positions compared with normal intervertebral disc heights, especially for the cross-sectional area and cranio-caudal foraminal height. The ventro-dorsal diameter showed an overall decrease between supine and upright positions, but less correlation with the grades of intervertebral disc narrowing. Additionally, our study showed that soft tissue image quality of the lumbar spine is significantly poorer using 3D tomography rather than using CT.

The lifetime prevalence of low back pain is about 84%, and the prevalence of chronic low back pain about 23%, with 11–12% of the population even being disabled by low back pain [31]. Thus, direct and indirect costs of low back pain cause a tremendous financial burden to the society [1]. According to the recent Global Burden of Disease Study and Global Spine (Phila Pa 1976) Care Initiative, lower back and neck pain are the largest contributors globally to years lived with disability from 1990 to 2015 and are ranked as the fourth leading cause of disability-adjusted life years [32, 33]. Therefore, cross-sectional examinations of the lumbar spine are common in addition to radiographs. However, these cross-sectional examinations are generally performed in the supine non-weight-bearing position, because upright MRI scanners are not widely available [2]. Additionally, weight-bearing CT examinations have only been feasible for the lower extremities, but not for the spine until now [3, 4].

The two spine units enabling motion of the spine are the facet joint and intervertebral space. Additionally, increasing lumbar lordosis and disc height change, especially in the aging spine, may be responsible for the dynamic changes of the foramina from the supine to upright position [7, 8]. Splendiani et al showed that the critical parameter for an “occult stenosis,” i.e., stenosis only visible in weight-bearing examinations, is the height of the intervertebral disc [14]. They found that stenosis of the neural foramen was never present in normal intervertebral disc height either in the presence or in the absence of facet degeneration, which points out the strong influence of the intervertebral disc height on the neural foramen in contrast to facet joint degeneration. Thus, we evaluated the neural foramina in our study regarding the height of the intervertebral disc.

All three parameters measured in our study demonstrate a significant position-dependent decrease of the neural foramina in the upright position. This is mostly in alignment with the study by Iwata et al who showed that foraminal height decreases for all three parameters at all lumbar segments except for the segment L5/S1 [8]. However, their healthy volunteer study group did not use a true upright position, but a simulation using a compression device. They suggested that a decreased pelvic angle, which occurs using a compressive device, widens segment L5/S1 [8]. Our upright examination showed an actual decrease in foraminal size at this level. This might be due to our study population, which consisted of patients with different grades of intervertebral disc degenerations. Mauch et al investigated the true standing position and also found changes for the lower lumbar segments L4/L5 and L5/S1, but not for the upper lumbar segments [5]. We believe this is because they used a grading system for foraminal changes rather than measuring the accurate diameter of the foramina. Thus, our approach might be more sensitive to delineate changes at all lumbar segments. Although all three parameters showed a decrease between supine and upright positions for all lumbar segments combined, this was not the case for observations of the individual level, especially for levels L4 and L5. Here one or both readers showed inconsistently higher values in the upright position. We believe this is attributed to the lower number of neural foramina included on these levels.

Our study shows that patients with higher grades of intervertebral disc height changes show a narrower neural foraminal size, especially for the cross-sectional area and cranio-caudal foraminal height. Interestingly, Hasegawa et al described in a cadaveric study a critical value for neuroforaminal stenosis of 15 mm cranio-caudal diameter [34]. Our study results show that in patients with intervertebral disc height reduction over 50%, this critical value is mostly surpassed. Additionally, in nearly all segments with less than 50% intervertebral disc height reduction, the cranio-caudal diameter was higher in the supine position, but reached the critical value in the upright position for some segments, e.g., level L3. This stresses out two points: (a) the influence of disc height changes on the size of neural foramina with possible compromisation of the nerve root, like presented in the study of Splendani et al [14], and (b) the possible underestimation of symptoms on supine imaging. The ventro-dorsal diameter correlated less with the grades of intervertebral disc narrowing. This is because intervertebral disc height changes are more pronounced in the cranio-caudal axis. Additionally, the higher interrater variability in ventro-dorsal diameters could be attributed to the low number of pixels compared with cross-sectional and cranio-caudal measurements. Measurements of an area include more pixels than measurements of a diameter. Therefore, statistical variations are more evident with pure diametric measurements, especially in small diameters, such as the ventro-dorsal diameter.

In our study, subjective image evaluation of the lumbar spine showed that twin robotic X-ray is inferior to CT. Twin robotic X-ray acquires 3D tomography in a cone-beam CT scanning mode. Studies of the lower extremity showed more artifacts and a lower image quality using 3D tomography compared with CT [25, 26, 28]. This is because CT scanners are better adjusted for corrections of beam-hardening and iterative reconstruction [35]. Benz et al investigated 3D tomography of the lumbar spine in five cadaveric specimens [24]. Their results showed an inferior image quality of 3D tomography compared with CT. Our first in vivo examinations confirm these results. A study by Demehri et al showed that cone-beam CT has a favorable bone but inferior soft tissue resolution compared with multidetector CT [36]. Since soft tissue depiction plays an important role in the diagnosis of nerve root affection, a detailed analysis of nerve pathologies might be limited as a result of the inferior soft tissue resolution using 3D tomography. This inferiority might be compensated by the use of intrathecal contrast administration using the fluoroscopic unit of this multifunctional system to assess the spinal canal and neural foramina in the upright weight-bearing position. But, intrathecal contrast administration is an invasive technique requiring specific indications and would most likely not be needed if upright MR imaging examination was broadly available. If indicated this intrathecal contrast administration might be of advantage in patients struggling to maintain the upright position, e.g., due to orthostatic syncope [10], to hold still for the length of MR examinations as image acquisition with 3D tomography with a duration of 40 s is comparatively short.

The duration of weight-bearing examinations performed with this unit is significantly shorter compared with MR in the supine and upright position, the latter usually has a longer scan time of each sequence, due to the low-field MR system which is mostly used for the upright, weight-bearing position [10]. One benefit of a 3D tomography is the short scan time, which may be more easily endured by patients with back pain or radiculopathy. Additionally, patients with non-conditional MR devices can be examined with 3D tomography in the upright, weight-bearing position. The twin robotic X-ray system provides also the possibility to perform radiographs and fluoroscopy. Thus, its application has the possibility for a broader clinical usage.

Limitations of our study are as follows: The scanning protocols in our study varied. This is due to scanning protocols adapted to the patient’s body mass index. Varying protocols, e.g., with or without copper filter, might have a direct impact on image quality. However, to reduce patients’ radiation exposure, we assumed the risk of image quality influence compared with the benefit of a lower radiation exposure as minimal. Nevertheless, to avoid higher impacts on image quality, we did not use copper filters with a thickness of more than 0.2 mm. We only measured the osseous diameters of the neural foramina and did not evaluate any nerve root restriction provoked by weight-bearing, i.e., due to the intervertebral disc or ligamentum flavum, because of inferior soft tissue contrast in 3D tomography compared with CT. Furthermore, low back pain or radiculopathy can also be related to a spinal canal or lateral recess stenosis, as well as degenerative changes of the intervertebral space or facet joints, and segmental instability. These were not examined in this study, and the clinical value of the twin robotic X-ray unit still needs to be evaluated for this purpose. Additionally, we did not correlate the image findings with clinical symptoms because the number of patients with clearly attributed nerve root symptoms was marginal. Fewer segments of the lower lumbar than of the upper spine were included because of the higher number of stabilizations performed on these levels. The artifacts caused by the metal implants led to primary exclusion of these segments.

## Conclusion

In the upright weight-bearing position, intervertebral disc height changes of varying extent occur, all leading to a smaller size of the lumbar foramina in comparison with supine position. Patients with narrower intervertebral disc height show a narrower neural foraminal size in supine and upright positions compared with normal intervertebral disc height, especially for the cross-sectional area and cranio-caudal foraminal height. Image quality, especially nerve root delineation, is inferior with the 3D tomography compared to CT.

## Abbreviations

3D: Three dimensional
CC: Cranio-caudal
cm: Centimeter
CSA: Cross-sectional area
CT: Computed tomography
kVp: Kilovolt peak
mAs: Milliampere-seconds
n: Number
sd: Standard deviation
VD: Ventro-dorsal
